# ^1^H, ^15^N, ^13^C backbone resonance assignments of human phosphoglycerate kinase in a transition state analogue complex with ADP, 3-phosphoglycerate and magnesium trifluoride

**DOI:** 10.1007/s12104-017-9758-3

**Published:** 2017-09-02

**Authors:** Zhalgas Serimbetov, Nicola J. Baxter, Matthew J. Cliff, Jonathan P. Waltho

**Affiliations:** 1 Manchester Institute of Biotechnology and School of Chemistry, The University of Manchester, 131 Princess Street, Manchester, M1 7DN UK; 2 Krebs Institute for Biomolecular Research, Department of Molecular Biology and Biotechnology, The University of Sheffield, Firth Court, Western Bank, Sheffield, S10 2TN UK

**Keywords:** Backbone resonance assignment, Transverse relaxation optimised spectroscopy, Phosphoryl transfer enzyme, Transition state analogue, Magnesium trifluoride

## Abstract

Human phosphoglycerate kinase (PGK) is an energy generating glycolytic enzyme that catalyses the transfer of a phosphoryl group from 1,3-bisphosphoglycerate (BPG) to ADP producing 3-phosphoglycerate (3PG) and ATP. PGK is composed of two α/β Rossmann-fold domains linked by a central α-helix and the active site is located in the cleft formed between the N-domain which binds BPG or 3PG, and the C-domain which binds the nucleotides ADP or ATP. Domain closure is required to bring the two substrates into close proximity for phosphoryl transfer to occur, however previous structural studies involving a range of native substrates and substrate analogues only yielded open or partly closed PGK complexes. X-ray crystallography using magnesium trifluoride (MgF_3_^−^) as a isoelectronic and near-isosteric mimic of the transferring phosphoryl group (PO_3_^−^), together with 3PG and ADP has been successful in trapping human PGK in a fully closed transition state analogue (TSA) complex. In this work we report the ^1^H, ^15^N and ^13^C backbone resonance assignments of human PGK in the solution conformation of the fully closed PGK:3PG:MgF_3_:ADP TSA complex. Assignments were obtained by heteronuclear multidimensional NMR spectroscopy. In total, 97% of all backbone resonances were assigned in the complex, with 385 out of a possible 399 residues assigned in the ^1^H–^15^N TROSY spectrum. Prediction of solution secondary structure from a chemical shift analysis using the TALOS-N webserver is in good agreement with the published X-ray crystal structure of this complex.

## Biological context

Phosphoglycerate kinase (PGK, EC 2.7.2.3) is a bisubstrate magnesium-dependent enzyme with highly conserved amino acid sequences found in all living organisms. In glycolysis, it catalyses the transfer of a phosphoryl group from 1,3-bisphosphoglycerate (BPG) to ADP producing 3-phosphoglycerate (3PG) and ATP, while in gluconeogenesis and in the Calvin cycle of photosynthetic species, PGK catalyses the reverse reaction. In mammals, PGK has further roles in oncogenesis and cellular metabolism, which have been exploited to provide targets for cancer treatments and retroviral infections. Elevated PGK concentrations in fibrosarcoma tumours promotes disulphide-bond reduction of plasmin resulting in a decrease in tumour vascularity, tumour growth and metastatic ability due to an increase in the plasma levels of angiostatin (an inhibitor of blood vessel development) (Lay et al. 2000). PGK activity in cell nuclei also modulates DNA replication, transcription and repair (Popanda et al. 1998) and this role has been utilised to generate and maintain equilibrium levels of l-nucleoside analogues (clinically active anticancer and antiviral treatments) through in vivo phosphorylation by PGK (Krishnan et al. 2003; Mathé and Gosselin 2006). There are two isoforms of human PGK which share 88% amino acid sequence identity: PGK1 is expressed in all cells, whereas PGK2 expression is confined to spermatogenic cells undergoing meiosis.

PGK is composed of two α/β Rossmann-fold domains linked by a central α-helix (F187−E201), with each domain comprising seven α-helices arranged around a six-stranded parallel β-sheet core together with a three-stranded antiparallel β-sheet insertion in the C-domain. The active site is located in the cleft formed between the N-domain which binds BPG or 3PG, and the C-domain which binds the nucleotides ADP or ATP. Several X-ray structures have been solved for PGK showing the domains in an open conformation [e.g. a PGK:ATP complex (Flachner et al. 2004)] and partly closed conformations [e.g. a PGK:3PG:ADP complex (Bernstein et al. 1997) and a PGK:3PG:AMP-PNP complex (Auerbach et al. 1997)], and a structural analysis (Zerrad et al. 2011) has proposed that the conformation of the enzyme in solution has a thermodynamic preference for the open form resulting from the protection from solvent of a hydrophobic patch located on the central α-helix.

Aluminium tetrafluoride (AlF_4_^−^), magnesium trifluoride (MgF_3_^−^) and transition metal oxoanion moieties have been used widely as transition state analogues (TSAs) of the trigonal planar PO_3_^−^ group in phosphoryl-transfer reactions (Wittinghofer 1997; Baxter et al. 2008, 2010; Jin et al. 2014, 2017; Wlodawer et al. 1983). Using metal-fluoride approaches, a fully closed human PGK:3PG:MgF_3_:ADP TSA complex was solved to a resolution of 1.47 Å (PDB: 2WZB; Cliff et al. 2010), in which clear electron density in the active site places the donor and acceptor oxygen atoms of 3PG and ADP (donor–acceptor distance = 4.3 Å) as apical ligands for a central trigonal bipyramidal MgF_3_^−^ group occupying the position of the transferring PO_3_^−^ group during the phosphoryl transfer reaction. The three equatorial fluoride atoms are hydrogen bonded by backbone amide groups, polar sidechains and the octahedrally coordinated catalytic Mg^II^ ion (F_1_ − K215:εNH_3_^+^ and Mg^II^; F_2_ − R38:ζNH_2_ and G373:HN; F_3_ − K219:εNH_3_^+^, G396:HN and H_2_O). Three discrete ^19^F resonances are clearly visible in a ^19^F NMR spectrum of this complex in solution (F_1_ = −160 ppm, F_2_/F_3_ = −153 and −156 ppm; Cliff et al. 2010) indicating that there is no significant intersite exchange on the millisecond timescale. In this work we report the ^1^H, ^15^N and ^13^C backbone resonance assignments of human PGK in the fully closed PGK:3PG:MgF_3_:ADP TSA complex, which represents the solution conformation of the X-ray crystal structure reported previously (PDB: 2WZB).

## Methods and experiments

### Protein expression and purification

A synthetic human PGK1 gene inserted into a pET-3a plasmid vector was purchased from GenScript. The plasmid was transformed into *Escherichia coli* strain BL21(DE3) cells (Stratagene) and ^2^H,^15^N,^13^C-labelled human PGK (43 kDa) was expressed in defined isotopically labelled minimal media, following the protocol of Reed et al. (2003). The cells were grown at 37 °C with shaking until OD_600nm_ = 0.8 and were induced by the addition of 1 mM isopropyl-β-d-thiogalactopyranoside (IPTG). After further incubation for 12 h, cells were harvested by centrifugation at 4000 rpm for 25 min at 4 °C. The cell pellet was resuspended in buffer A (10 mM sodium phosphate buffer pH 6.2, 1 mM DTT, 1 mM EDTA) supplemented with cOmplete™ protease inhibitor cocktail (Roche) (one tablet per 50 mL of buffer). The cell suspension was lysed on ice by sonication for 12 cycles of pulsation for 15 s with 45 s cooling intervals. The cell lysate was then separated by ultracentrifugation at 39,800×*g* (17,000 rpm) for 25 min at 4 °C in a Beckman Coulter Avanti J–E centrifuge using rotor JA 17. Ammonium sulphate was added slowly with gentle agitation to the supernatant to 40% saturation (246 g L^−1^). Following complete solubilisation of the ammonium sulphate, the solution was centrifuged as before to pellet unwanted insoluble proteins. The supernatant was removed carefully and ammonium sulphate was added slowly with gentle agitation to 80% saturation (284 g L^−1^) to precipitate PGK, which was pelleted as before by centrifugation. The pellet was resuspended carefully in buffer A and the protein solution was treated with 10 mg mL^–1^ protamine sulphate (Sigma-Aldrich) for 1 h to precipitate nucleotides, which were removed by centrifugation. The supernatant was filtered using a 0.22 μm syringe filter (Sartorius Mechatronics UK Ltd.) and was dialysed against 4 L of buffer A for 6 h at 4 °C, followed by a further 4 L of buffer A for an additional 6 h. The protein sample was filtered through a 0.22 µm syringe filter and loaded onto a CM-Sepharose cation exchange column (Sigma-Aldrich) connected to an ÄKTA purification system (GE Healthcare) that had been washed previously with 5 column volumes of filtered and degassed buffer A, followed by 5 column volumes of filtered and degassed buffer B (buffer A containing 1 M NaCl) and equilibrated with at least 5 column volumes of filtered and degassed buffer A. Proteins bound to the CM-Sepharose column were eluted with a gradient of 0 to 100% buffer B. PGK elutes as a well-defined peak at around 20% buffer B. Fractions containing PGK were checked for purity by SDS–PAGE, were pooled together and were concentrated by Vivaspin (10 kDa MWCO, GE Healthcare). Once purified, back exchange to amide protium atoms in perdeuterated PGK was promoted by an unfolding-refolding process. The PGK sample was exchanged into buffer C (50 mM Tris–HCl buffer pH 7.4, 5 mM MgCl_2_, 20 mM DTT, 1 mM EDTA) by repeated dilution-concentration steps (three times) by Vivaspin. Protein unfolding was initiated by mixing the sample with buffer C containing 6 M GuHCl, in a ratio such that the final concentration of GuHCl was 2.9 M. Protein refolding was achieved by rapid 30-fold dilution of the PGK sample into buffer C. Finally, the PGK solution was concentrated to NMR sample concentrations by Vivaspin and dialysed three times against 1 L of buffer C for 4 h at 4 °C, to remove GuHCl from the unfolding step. Protein concentrations were estimated by absorbance at 280 nm (ε_280_ = 27,960 M^− 1^ cm^− 1^). All reagents including the stable isotopically-labelled compounds ^15^NH_4_Cl (99%), ^13^C_6_,^2^H_7_-d-Glucose (U–^13^C_6_, 99%; 1,2,3,4,5,6,6-d_7_ 97–98%) and ^2^H_2_O (99.8%) were purchased with the highest purity from Sigma-Aldrich and used as received.

### NMR spectroscopy

All NMR experiments were recorded at 298 K, using standard pulse sequences on an 800 MHz Bruker Avance III NMR spectrometer equipped with a TCI cryoprobe, Z gradients and TopSpin software version 3.2, housed in the Manchester Institute of Biotechnology. NMR samples containing 0.5 mM ^2^H,^15^N,^13^C-labelled human PGK, 10 mM 3PG, 10 mM ADP, 10 mM MgCl_2_, 30 mM NH_4_F in 50 mM Tris–HCl buffer pH 7.4, 20 mM DTT, 1 mM EDTA and 0.04% v/v NaN_3_ were loaded into 5-mm diameter NMR tubes. ^2^H_2_O (10% v/v) and trimethylsilyl propanoic acid (TSP; 0.5% v/v) were added to the NMR samples for the deuterium lock and as a chemical shift reference, respectively. ^1^H chemical shifts were referenced to the internal TSP signal, whereas ^15^N and ^13^C chemical shifts were referenced indirectly using nuclei-specific gyromagnetic ratios. For the backbone ^1^H, ^15^N and ^13^C resonance assignment, standard Bruker ^1^H–^15^N TROSY and TROSY-based 3D versions of HNCA, HNCACB, HN(CO)CACB, HN(CA)CO and HNCO experiments were acquired using non-uniform sampling with a multidimensional Poisson Gap scheduling strategy with sinebell weighting (Hyberts et al. 2013). A 30 Hz (0.15 ppm) resolution in the ^13^C dimension was obtained after processing. The HNCO spectrum, with one peak per residue in the ^13^C dimension was obtained with 230 hypercomplex points, whereas spectra with two peaks per residue [HNCA, HN(CO)CACB, HN(CA)CO] were obtained with 460 hypercomplex points and the HNCACB spectrum with four peaks per residue was obtained with 920 hypercomplex points.

### Resonance assignments and data deposition

Backbone ^1^H_N_, ^15^N, ^13^C_α_, ^13^C_β_ and ^13^C′ resonances were assigned for human PGK in the PGK:3PG:MgF_3_:ADP TSA complex using standard triple resonance methodology (Gardner and Kay 1998). Spectra were processed with TopSpin software version 3.2. Peak picking and frequency matching was performed within CcpNmr Analysis version 2.4 (Vranken et al. 2005) and the backbone assignments were confirmed independently using a simulated annealing algorithm employed by the “asstools” assignment program (Reed et al. 2003). The backbone ^1^H, ^15^N and ^13^C chemical shifts have been deposited in the BioMagResBank (http://www.bmrb.wisc.edu/) under the BMRB accession code 27022. The human PGK1 construct used in this study results in the PGK protein sequence M1-S2-L3-S4-N5……I417, whereas the residue numbering used in the X-ray crystal structure of the PGK:3PG:MgF_3_:ADP TSA complex is defined as S1-L2-S3-N4……I416. For clarity and ease of comparison, the crystal structure nomenclature has been used here throughout.

**Fig. 1:**
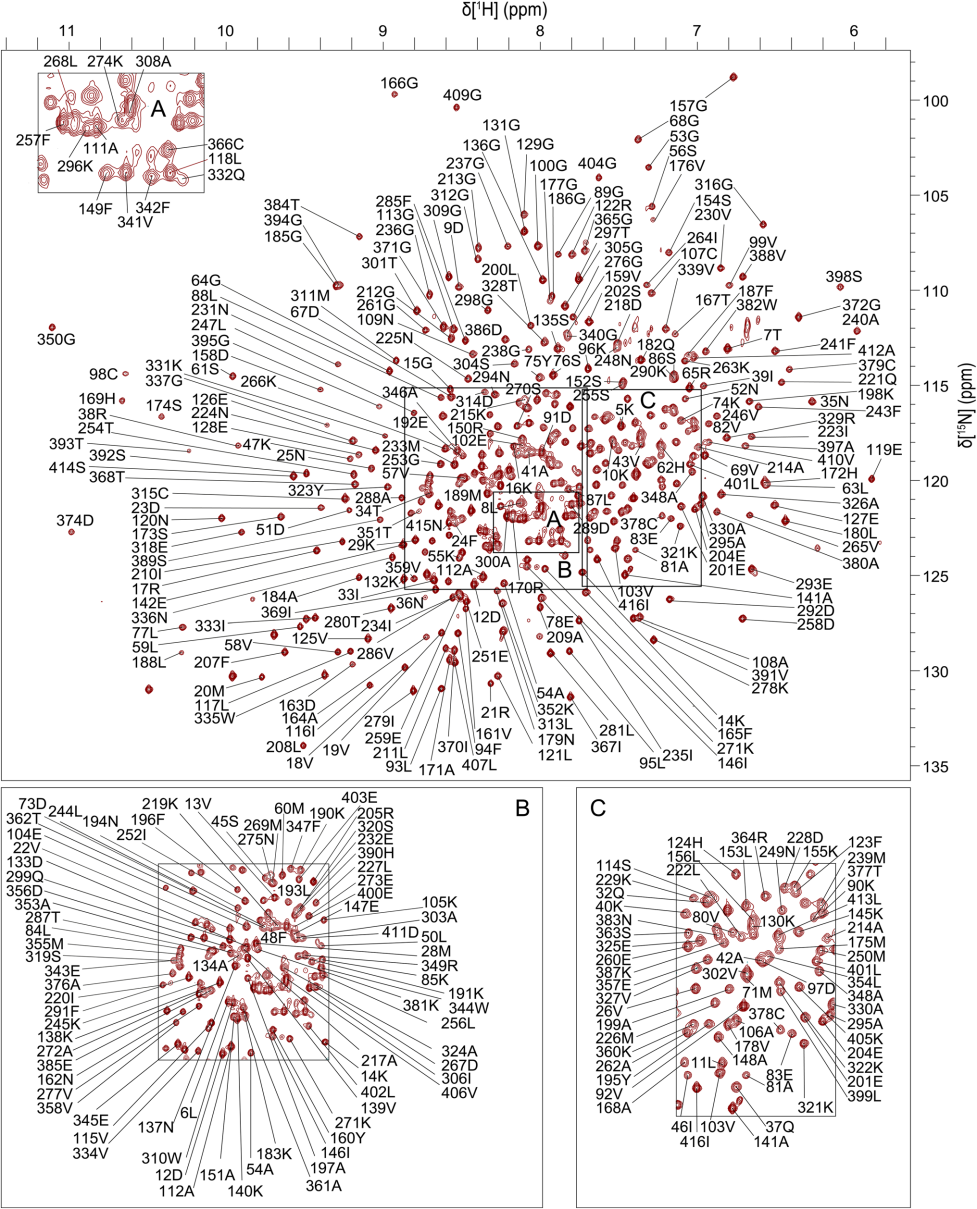
^1^H–^15^N TROSY spectrum of the PGK:3PG:MgF_3_:ADP TSA complex recorded at pH 7.4 and 298 K. The assignments of backbone amide resonances are indicated by sequence number and residue type as defined in the crystal structure (PDB: 2WZB)

**Fig. 2:**
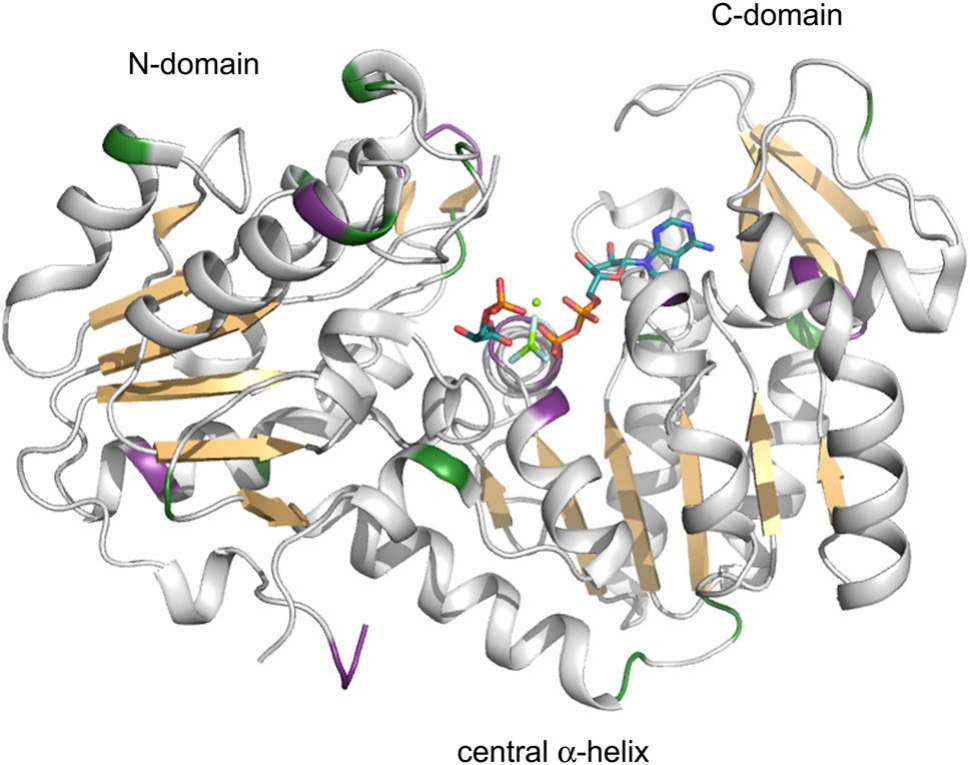
Cartoon representation of the crystal structure of the PGK:3PG:MgF_3_:ADP TSA complex showing the extent of backbone amide resonance assignments. Assigned residues are coloured *white* and *tan*, proline residues are coloured *green* and unassigned residues are coloured *purple*. The ligands 3PG, MgF_3_^−^ and ADP are indicated as CPK-coloured sticks and the catalytic magnesium ion is shown as a *green sphere*. For clarity, the β-strands have been coloured *tan* to highlight the domain structure of PGK

Excluding the 17 proline residues and the N-terminal methionine from the 417-residue PGK protein sequence, 385 out of a total of 399 residues were assigned in the ^1^H–^15^N TROSY spectrum of the PGK:3PG:MgF_3_:ADP TSA complex (Fig. 1). In total, 97% of all backbone resonances were assigned (96% of ^1^H_N_, 96% of ^15^N, 98% of ^13^C_α_, 98% of ^13^C_β_ and 98% of ^13^C′ nuclei). There are 14 residues that remain unassigned in the ^1^H–^15^N TROSY spectrum (S1, L2, S3, N4, N30, N31, C49, A144, V216, T242, V283, G373, T375 and G396) and their ^1^H-^15^N TROSY correlations are likely to be attenuated beyond detection by either fast exchange with solvent or intermediate exchange broadening on the millisecond timescale (Fig. 2). For the backbone amide groups of both G373 and G396, an additional source of signal attenuation arises from 1-bond ^1^H−^19^F coupling and 2-bond ^15^N−^19^F coupling through hydrogen bonding to F_2_ and F_3_ respectively of the MgF_3_^−^ moiety (Baxter et al. 2010).

**Fig. 3:**
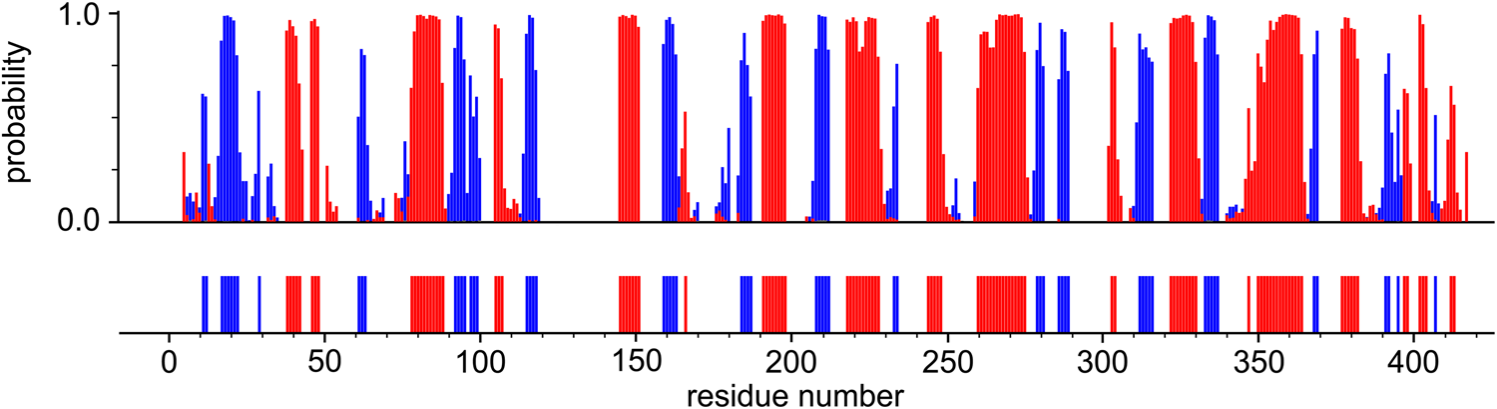
Secondary structure prediction of PGK in the PGK:3PG:MgF_3_:ADP TSA complex obtained with TALOS-N (Shen and Bax 2013) using the backbone ^1^H_N_, ^15^N, ^13^C_α_, ^13^C_β_ and ^13^C′ chemical shifts (*upper panel*). The secondary structure prediction is shown as *red bars* for α-helices and *blue bars* for β-strands, with the height of the bars representing the probability assigned by the software. The secondary structure derived from the crystal structure of the PGK:3PG:MgF_3_:ADP TSA complex is reported in the *same colour* representation (*lower panel*)

The secondary structure content of PGK was predicted by uploading the backbone ^1^H_N_, ^15^N, ^13^C_α_, ^13^C_β_ and ^13^C′ chemical shifts of the PGK:3PG:MgF_3_:ADP TSA complex to the TALOS-N webserver (Shen and Bax 2013). Figure 3 illustrates the comparison between the predicted secondary structure for the solution PGK:3PG:MgF_3_:ADP TSA complex and the secondary structure present in the crystal. These data are in very good agreement, which indicates that the solution conformation is similar to the protein structure observed in the crystal, and provides confidence in the assignments of the PGK:3PG:MgF_3_:ADP TSA complex.
